# A comparative study utilizing finite element modeling to analyze the biomechanical differences in various cement distributions within osteoporotic vertebral compression fractures

**DOI:** 10.1097/MD.0000000000049754

**Published:** 2026-07-17

**Authors:** Changbing Wu, Jie Liu, Hao Long, ZhuBo He, Fu Yong, Guo Xian Wang, Ge Bing, Hai Tao Gao

**Affiliations:** a Department of Spinal Surgery, Guiyang Fourth People’s Hospital, Guiyang, China.

**Keywords:** biomechanics, bone cement, finite element analysis, osteoporotic vertebral compression fractures, percutaneous vertebroplasty

## Abstract

This study aims to assess the biomechanical impact of bone cement perfusion on osteoporotic vertebral compression fractures across various distribution sites through finite element simulation. The finite element model of the L1–L3 vertebral body was constructed and its efficacy was validated. Simulated compression fractures of the L2 vertebral body resulted in the distribution of cement in the anterior, middle, and posterior thirds of the L2 vertebral body. Physiological loading and boundary conditions of axial compression experienced by the spine in the upright position were established, and loading models were examined in various states of movement including forward flexion, extension, left and right lateral bending, and left and right rotational movements. The study compared the stress distribution, maximum Von-Mises stress, maximum elastic strain of the vertebral body, and maximum deformation displacement of the vertebral body and intervertebral disc based on the distribution differences of L2 vertebral bone cement. In flexion, extension, and lateral bending, the peak stress experienced by the L2 vertebral body due to the bone cement model was found to be lower in the anterior 1/3 compared to the middle and posterior 1/3. Furthermore, when the left side of the vertebral body was subjected to bending and rotation, the maximum stress values observed in the fracture model and the posterior 1/3 of the L2 vertebral body cement model on the L3 vertebral body were similar to, and significantly higher than, those seen in the anterior and middle 1/3 of the L2 vertebral body cement models. The elastic modulus of the vertebral body is enhanced when bone cement is strategically placed in the anterior middle region, potentially facilitating the transmission of pressure from the vertebral body to the upper and lower lumbar spine through the bone cement. This redistribution of pressure may alleviate stress in the central region, thereby reducing the likelihood of vertebral injury and adjacent vertebral fractures.

## 1. Introduction

Osteoporotic vertebral compression fractures are prevalent in over 30% of individuals aged 65 years and older, with a higher incidence observed with advancing age.^[1,2]^ This particular type of fracture is more common than other osteoporotic fractures and is associated with a range of debilitating consequences such as low back pain, spinal deformity, loss of spinal function, decreased quality of life, and mortality. Consequently, osteoporotic vertebral compression fractures have emerged as a significant global health concern.^[2–5]^ Percutaneous vertebroplasty (PVP) is a commonly utilized intervention for these fractures, aimed at alleviating pain and restoring spinal function through the stabilization, support, and thermal destruction of nerve endings with bone cement.^[6,7]^ The benefits of this technique include reduced trauma, shorter operation time, quicker recovery, and early resumption of the patient daily activities.^[4,8]^ Nevertheless, the widespread adoption of percutaneous vertebroplasty technology has brought to light concerns regarding heightened risks of adjacent vertebral fractures, re-collapse of the reinforced vertebral body, and excessive financial burdens.^[9,10]^ The Pezeshki PS study^[11]^ suggests that varying distribution regions of cement within the vertebral body biomechanically influence the likelihood of adjacent vertebral fractures following PVP. Several retrospective studies have indicated that factors such as cement perfusion, postoperative anti-osteoporosis treatment, and cement leakage may contribute to postoperative refractures of the affected vertebra or neighboring vertebral bodies. However, the current body of evidence-based medical literature is deemed insufficient to definitively establish these relationships.^[12,13]^ Because there are few studies on the distribution and refracture of bone cement, this study investigated the modular distribution of bone cement from finite element and biomechanical point of view to explore the performance of spinal biomechanics in different distribution states. Due to the limited existing research on the distribution and refracture of bone cement, this study examined the modular distribution of bone cement from both finite element and biomechanical perspectives in order to assess the impact on spinal biomechanics in various distribution states. Finite element analysis, a valuable tool in computational mechanics, offers cost-effectiveness, repeatability, and accuracy, making it a widely utilized method in biomechanical studies within the field of orthopedics.^[14–16]^ It is widely accepted that the finite element model (FEM) of the spine is a valuable tool for assessing spinal injuries from a biomechanical perspective.^[17]^ In this study, a three-dimensional FEM of the L1–L3 vertebral body was developed, with L2 designated as the fractured vertebra. The ideal position for bone cement injection was determined to simulate the movement of the lumbar spine under 6 different conditions: forward flexion, extension, left and right scoliosis, and left and right rotation. The study aimed to investigate the impact of varying distribution positions of PVP bone cement on the injured vertebra body and adjacent vertebral body structures. The objective of this study was to investigate the variations in structural biomechanical properties of the vertebral body resulting from different positions of cement distribution following PVP, with the intention of offering a theoretical framework for surgeons to enhance surgical outcomes and minimize complications.

In this study, tetrahedral meshes were adopted to simulate bone tissue. Body weight and lumbar spine movements were applied as physical loads. Meanwhile, simplified material properties were assigned to human ligaments, cartilage, intervertebral discs (IVD) and other structures according to their Poisson ratios. All these factors may influence the research results. Therefore, the findings of this study only serve as a reference reminder and cannot yet be directly and definitively applied as clinical guidance.

## 2. Materials and methods

### 2.1. Major equipment

Siemens 128-slice CT machine (MIMICS 21.0 Mate rialise, Germany); Geomagic Studio2017 (Geomagic, USA); Solidworks2022 (Solidworks, USA); Workbench 2020 R2 (Anasys, USA).

### 2.2. Study subjects and establishment of three-dimensional model of L1-3 vertebral body

An elderly female patient, who was previously healthy and had no history of spine-related diseases or trauma, underwent thin-section CT scanning of the lumbar spine with a slice thickness of 1 mm to obtain images in DICOM format. The CT images were imported into Mimics 19.0 software to optimize the CT images. After clicking Calculate 3D to generate the corresponding model of L1-3 vertebral body and performing simple operations such as filling obvious cavities, Calculate 3D is clicked again to generate a three-dimensional model to obtain the point cloud data of the lumbar spine. L1–3 vertebral models were stored separately in STL format.

### 2.3. Optimization of three-dimensional models of the L1-3 vertebral body

The L1–3 vertebral body model in STL format was imported into Geo-magicstudio2017 software at the same time to hide the L2–3 vertebral body, and the L1 model was treated with polygonal function to remove features, fill cavities, and smooth. Use the “Grid Doctor” check in Geomagicstudio2017 software until the analysis items are all 0 for accurate curved surface processing. Detect contour line in precise surface processing, further edit contour line, and construct surface film after editing. Check that no paths intersect to construct the grid, and generate the fitting surface after the construction is completed. Copy L1 vertebral body model, hide L1 model, open the copy, transform it into polygonal module, after all selection, select “select offset.” After filling the cavity, it was performed again according to the L1 vertebral body processing step until the fitting surface was completed and stored separately in STEP format. After completing the L2–3 vertebral body and its duplicates according to the above steps, they were saved to the same folder. At this time, the initial model of cortical and cancellous bone of L1–3 vertebral body was formed.

### 2.4. Model assembly

**Figure 1.**
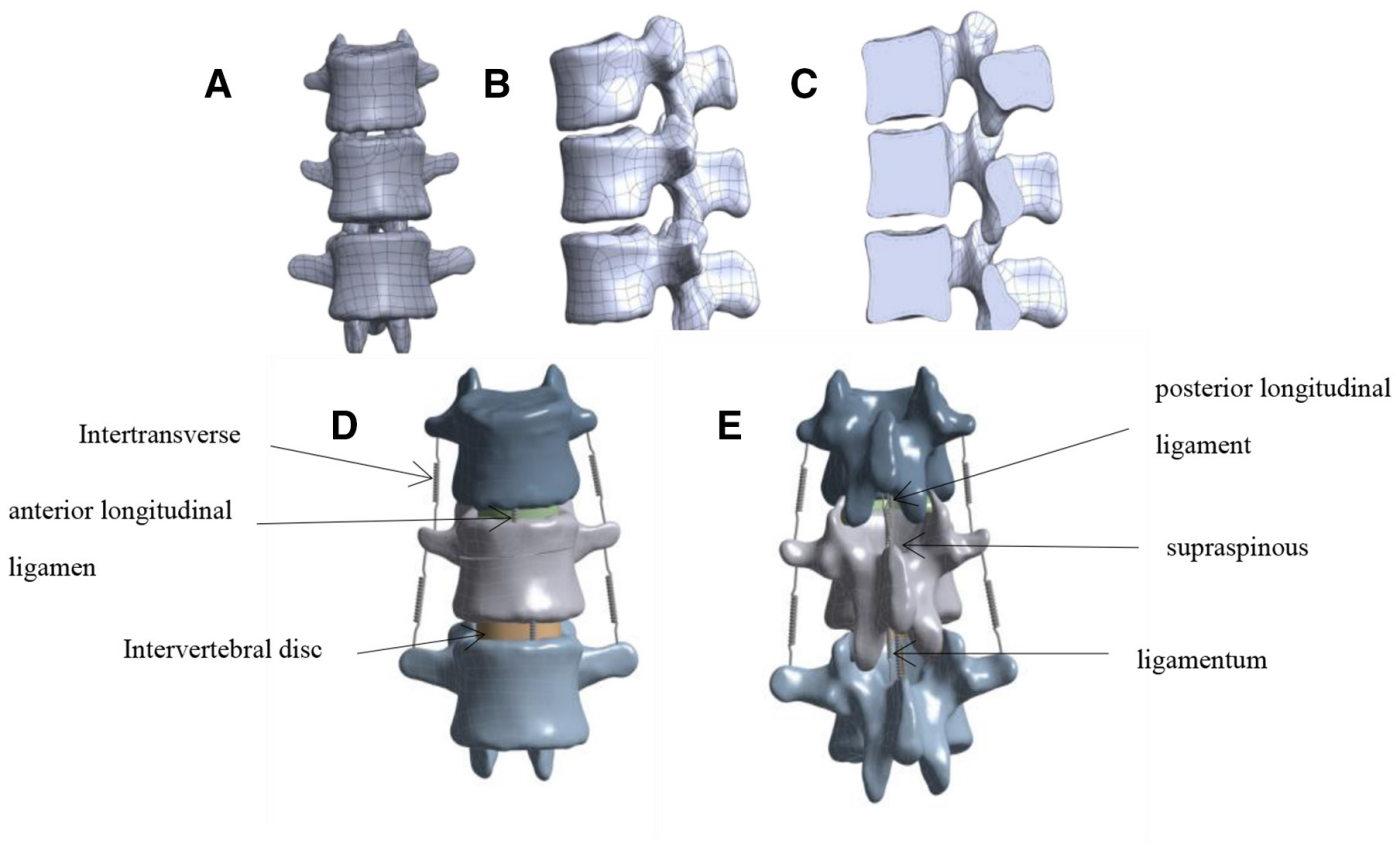
Establishment of lumbar 1 to 3 vertebral body model and assignment of materials for each tissue. (A) Anterior view of L1-3 vertebral body model. (B) Left view of L1-3 vertebral body model. (C) Cross-sectional view of the L1-3 vertebral body model. In Ansys, the finite element models of each tissue material of L1-3 vertebral body were assigned, including intervertebral disc and ligament structures. (D) Anterior view of the model after assignment of each tissue of the L1-3 vertebral body; (E) Anterior view of the model after assignment of each tissue of the L1-3 vertebral body.

The 6 generated STEP files were imported into the Solid works2017 software at the same time, and the 6 models were assembled together. After assembly, L1–3 cortical bone was hidden, 3 vertebral cancellous bones were duplicated, and the cortical bone was combined with duplicated cancellous bone by clicking the combination, respectively, and cancellous bone was removed to complete the establishment of the final model of cortical cancellous bone. The established cortical cancellous bone was then stored separately in assembly and component formats. After the completion of storage, open the cortical cancellous bone in the form of component again. Hide the cortical cancellous bone of L1 vertebral body, use the upper surface of L2 vertebral body as the base plane, use the sketch curve tool to draw the IVD contour along the edge of upper surface of vertebral body, then click the stretching convex table in the characteristics, select the 2 sides of symmetrical and uncombined results, and adjust the convex table height to 30 mm. Hidden convex table, 0 mm equidistant L2 vertebral body upper surface and L1 vertebral body lower surface, respectively, generate curved surface 1 and curved surface 2. A preliminary model of IVD was obtained by dividing the convex table with 2 equidistant surfaces and removing the convex table on both sides of the surface. Curve 1 and surface 2 were divided into surface 3 and surface 4, and the remaining convex tables were divided again with surface 3 and surface 4 to generate the endplates on both sides. Hide both endplates and all curved surfaces, display the reference plane 1 again, use the curve tool in the sketch to draw the outline of nucleus pulposus in the remaining convex table, use the sketch to segment the convex table in direct editing, generate the nucleus pulposus model, the nucleus pulposus area accounts for about half of the IVD,^[16]^ and obtain the L1-2 IVD and nucleus pulposus model. Then complete the L2-3 IVD and nucleus pulposus establishment according to the above steps and save them to the same folder, adopting the stepwise strategy of datum plane—sketch—surface splitting enables precise control over the spatial dimensions and relative positions of each IVD structure, and solves the difficulties in accurate modeling of complex curved surface structures. At this time, the initial model of nucleus pulposus of L1-2 and L2-3 IVDs was formed. Cortical bone, cancellous bone and nucleus pulposus models of L1-3 vertebral body were imported into Workbench 2020 R2 software. The format compatibility between SolidWorks and Workbench ensures seamless conversion from geometric models to FEMs, reducing the geometric repair workload during meshing. Spring units were used to simulate the main ligaments such as anterior longitudinal ligament, posterior longitudinal ligament, ligamentum flavum, supraspinous ligament and intertransverse process. The use of spring elements to simulate ligaments and the differentiated definition of contact types simplifies computational complexity while retaining the core influencing factors of spinal biomechanical responses, which conforms to the engineering application principles of finite element analysis (Fig. 1). Click Engineering Data to input the material properties of each structure of spine^[18,19]^ according to the indicators in Table 1, close this window after input, click Model option to assign the corresponding materials. The facet joint surface was defined as Frictional, the coefficient of friction was set to 0.1,^[20]^ and the remaining contact surfaces were defined as Bonde by default.

**Table 1:** Material properties of various spinal structures and main ligaments.

Material	Elastic modulus(Mpa)	Cross-sectional area(mm^2^)	Poisson ‘s ratio
Cortical bone	8040	–	0.30
Spongy bone	34	–	0.20
PMMA Bone cement	3000	–	0.40
Nucleus pulposus	1	–	0.50
Endplate	24	–	0.25
Annulus fibrosus	4	–	0.45
Cartilage	10	–	0.4
ALL	8	22	0.3
PLL	10	7	0.3
LF	17	14	0.3
LL	10	0.6	0.3
SL	8	10.5	0.3

– = No cross-sectional area, ALL = Anterior longitudinal ligament, LF = Ligamentum flavum, LL = Intertransverse ligament, PMMA = Polymethylmethacrylate, PLL = Posterior longitudinal ligament, SL = Supraspinous ligament.

### 2.5. Model of L2 vertebral body fracture and bone cement distribution in different locations

**Figure 2.**
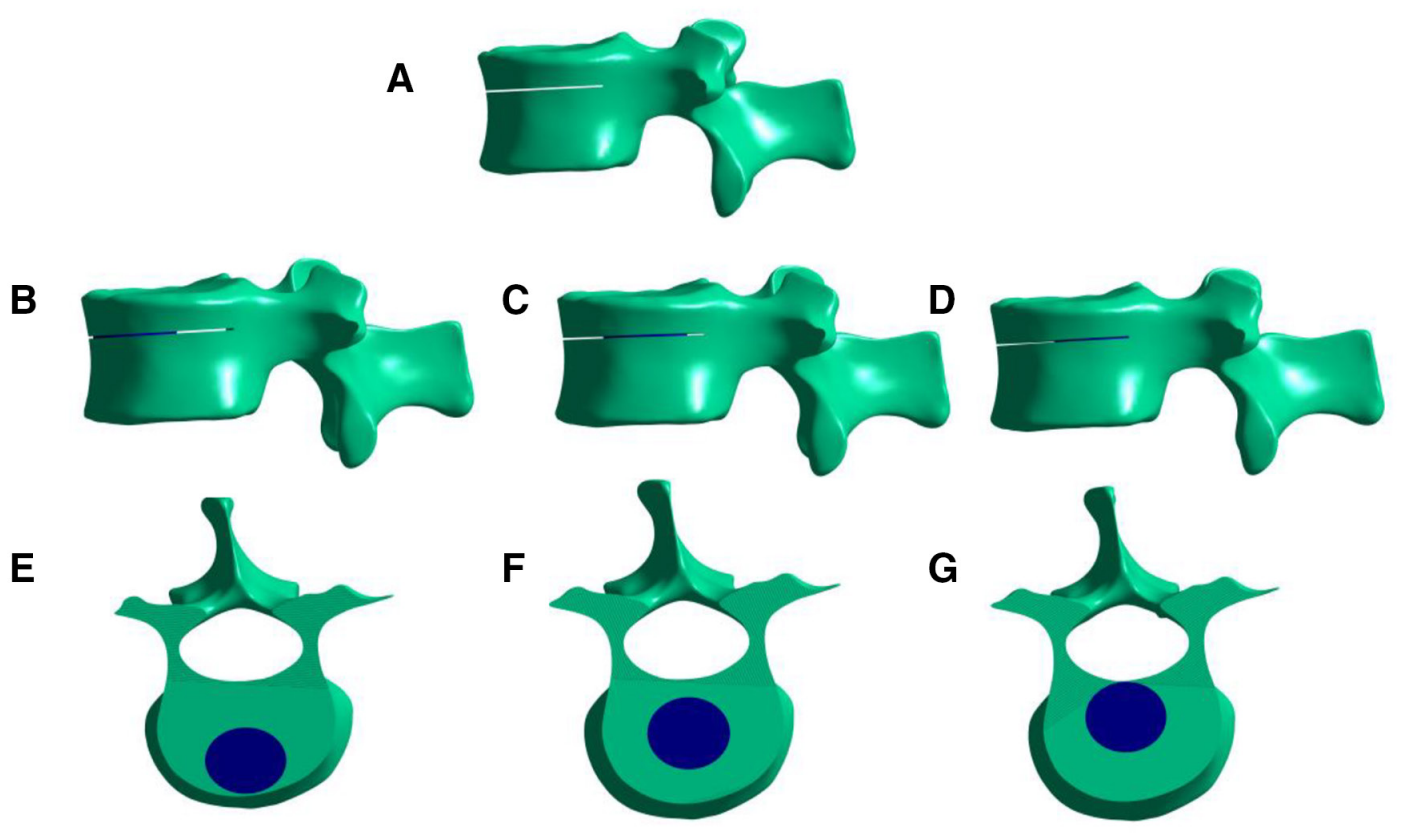
Simulated L2 vertebral fracture model and injected cement position. (A): L2 vertebral fracture model (without bone cement perfusion); (B, E): bone cement perfusion in the anterior 1/3 of the lumbar 2 vertebral fracture vertebral body (lateral and prone views); (C, F): bone cement perfusion in the middle 1/3 of the lumbar 2 vertebral fracture vertebral body (lateral and prone views); (D, G): bone cement perfusion in the posterior 1/3 of the lumbar 2 vertebral fracture vertebral body (lateral and prone views).

The L2 vertebral fracture model was simulated by solid works 2017 software by cutting the cortical and cancellous bone of the vertebral body to form a fracture line that horizontally penetrates the vertebral body by 24.1 mm, with a width and height of approximately 43.6 mm and 0.5 mm, respectively.^[21,22]^ The fracture model was imported into Solidworks software. The cement injection model was created by the software using a 4 mL straight post similar to the cement model.^[23]^ The cement model was perfused and distributed in the anterior, middle and posterior 1/3 of the L2 vertebral body using the assembly command, and then the excess bone was removed by Boolean operation to assemble the cement model into the PVP vertebral body. At this time, we obtained L2 vertebral fracture unperfused cement model, L2 vertebral anterior 1/3 perfused cement model, L2 vertebral middle 1/3 perfused cement model and L2 vertebral posterior 1/3 perfused cement model (Fig. 2).

### 2.6. Boundary and loading conditions for FEMs

**Figure 3.**
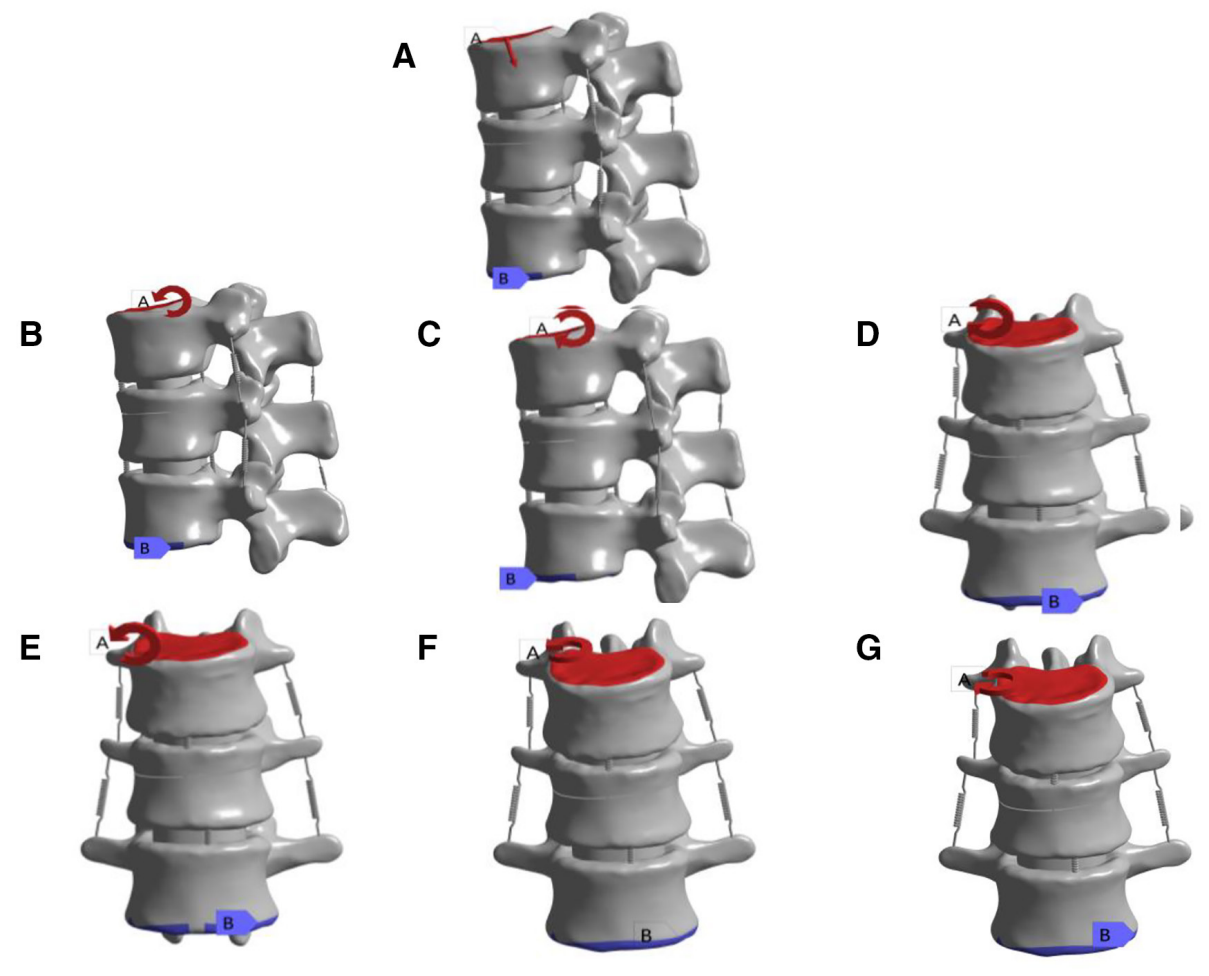
Boundary and loading conditions for the finite element model of the lumbar 1 to 3 vertebral body. (A) A represents that a continuous downward physiological load of 500 N is continuously applied to the upper surface of the L1 vertebral body, B represents that the lower surface of the L3 vertebral body is completely fixed; (B–G) a continuous downward physiological load of 500 N is continuously applied to the upper surface of the L1 vertebral body and the lower surface of the L3 vertebral body is completely fixed at the same time, pure moments of 10 N/m are applied to the anteroinferior, posteroinferior, left lower, right lower, left axial and right axial directions of the L1 vertebral body surface to simulate 6 working conditions: vertebral flexion, extension, left bending, right bending, left rotation, and right rotation.

The assembly model is meshed, and tetrahedral meshes are generated by bone tissue. After completion, the lower surface of L3 vertebral body was constrained and fixed by Static Structural in Workbench 2020 R2 software, and the upper surface of L1 vertebral body was subjected to 500N axial compressive load to simulate the weight of upper half segment of human body.^[24]^ Flexion-extension, left-right lateral bending, and left-right axial rotation were performed using pure moments of 10 Nm and 500 N pre-compressive loads (Fig. 3). First, the validity of the model was verified by measuring the range of motion of the lumbar spine. The distribution and maximum value of von-Mises stress on L1-2 and L2-3 IVDs and L1-3 vertebral bodies were then calculated.

## 3. Results

### 3.1. Validation of model validity

**Figure 4.**
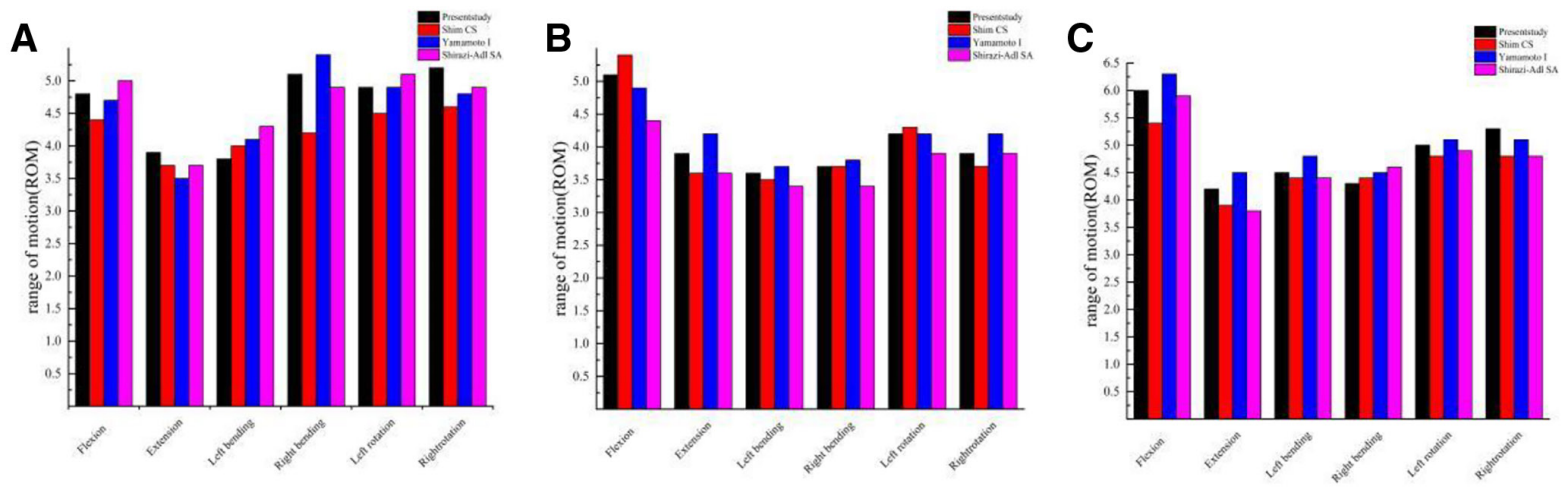
L1-3 Vertebral Body Model Validation. (A) ROM of L1-2 under 6 loading conditions; (B) ROM of L2-3 under 6 loading conditions; (C) ROM of L1-3 under 6 loading conditions.

Compared with the previous experimental biomechanical results,^[25–27]^ the range of motion of this experimental model was within the normal range under each of the 6 working conditions (forward flexion, extension, left lateral bending, right lateral bending, left rotation, and right rotation) (Table 2, Figure 4), demonstrating that the model established in this study was effective.

**Table 2:** ROM of L1-2, L2-3, L1-3 compared to published literature.

Vertebral	L1-2			L2-3					L1-3					
Variabe	Presentstudy	Shim CS^[22]^	Yamamoto I^[23]^		Shirazi-Adl SA^[24]^	Present study	Shim CS^[22]^	Yamamoto I^[23]^		Shirazi-Adl SA^[24]^	Present study	Shim CS^[22]^	Yamamoto I^[23]^	Shirazi-Adl SA^[24]^
Flexion	4.8	4.4	4.7		5.0	5.1	5.4	4.9		4.4	6.0	5.4	6.3	5.9
Extension	3.9	3.7	3.5		3.7	3.9	3.6	4.2		3.6	4.2	3.9	4.5	3.8
Left bending	3.8	4.0	4.1		4.3	3.6	3.5	3.7		3.4	4.5	4.4	4.8	4.4
Right bending	5.1	4.2	5.4		4.9	3.7	3.7	3.8		3.4	4.3	4.4	4.5	4.6
Left rotation	4.9	4.5	4.9		5.1	4.2	4.3	4.2		3.9	5.0	4.8	5.1	4.9
Rightrotatio	5.2	4.6	4.8		4.9	3.9	3.7	4.2		3.9	5.3	4.8	5.1	4.8

### 3.2. Distribution and magnitude of von-Mises stress on L2 vertebral body

**Figure 5.**
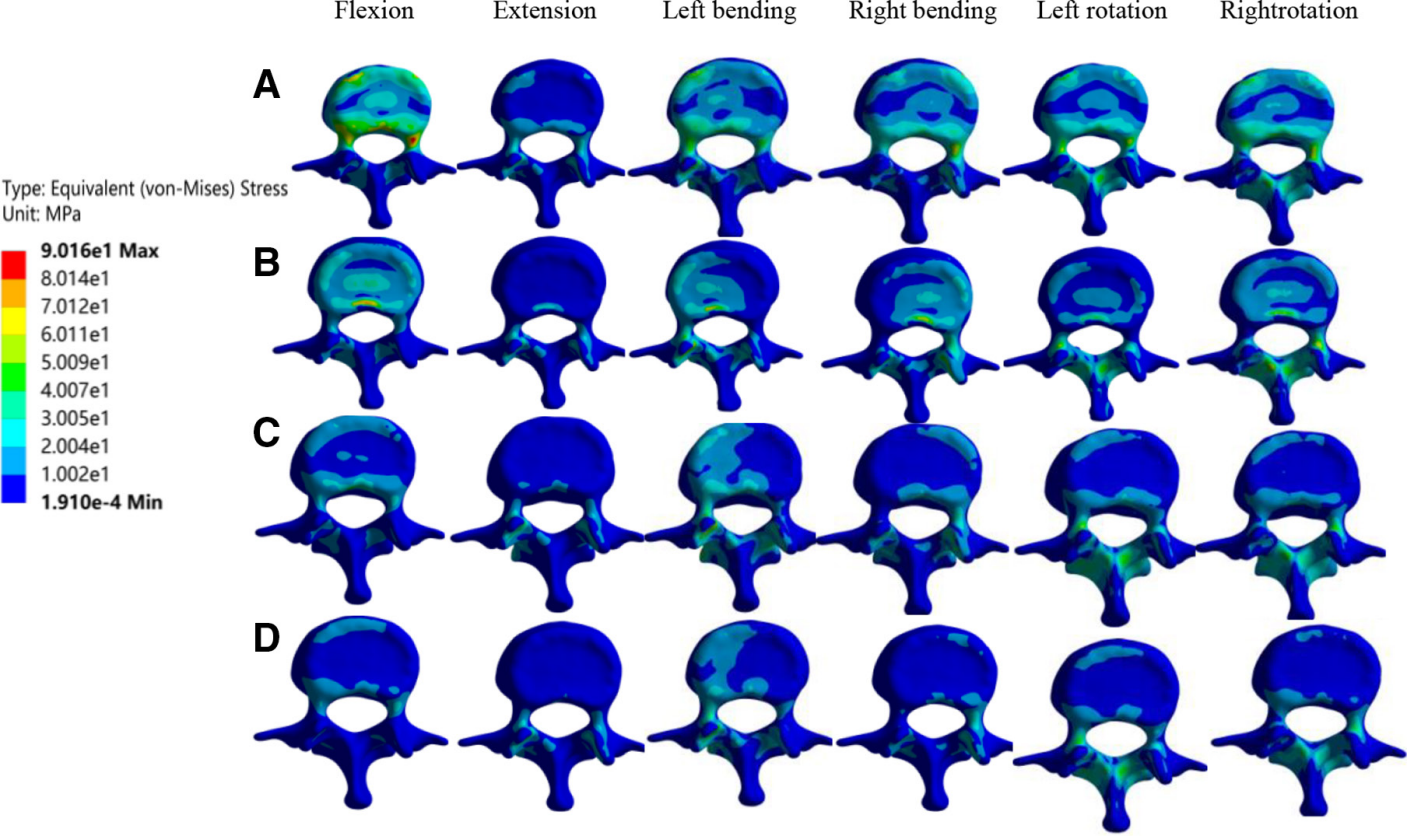
Stress distribution under 6 working conditions in L2 vertebral fracture unperfused cement model, anterior 1/3, middle 1/3, and posterior 1/3 cement models. (A) Stress distribution when bone cement is not perfused in L2 vertebral fracture; (B) Stress distribution when bone cement is perfused in the anterior 1/3 of L2 vertebral fracture; (C) Stress distribution when bone cement is perfused in the middle 1/3 of L2 vertebral fracture; (D) Stress distribution when bone cement is perfused in the posterior 1/3 of L2 vertebral fracture.

**Figure 6.**
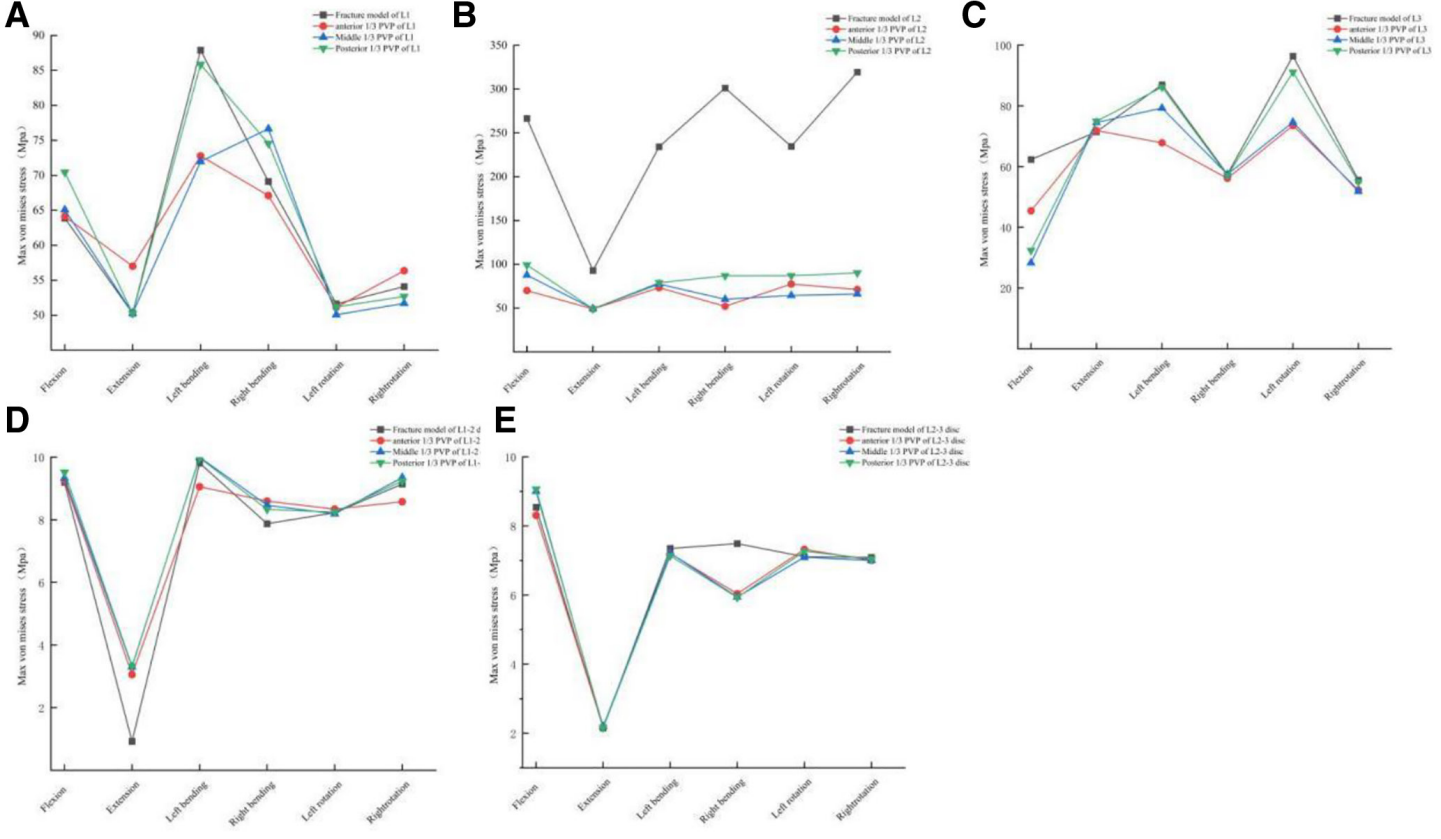
Maximum von-Mises stress diagram for each vertebral body and disc. (A) Maximum stress map of L1 vertebral body under 6 working conditions (flexion and extension, left and right lateral bending, left and right rotation) of L2 vertebral body fracture, anterior 1/3 PVP, middle 1/3 PVP and posterior 1/3 PVP models; (B) Maximum stress map of L2 vertebral body under 6 working conditions: L2 vertebral fracture, anterior 1/3 PVP, middle 1/3 PVP and posterior 1/3 PVP models; (C) Maximum stress map of L3 vertebral body fracture, anterior 1/3 PVP, middle 1/3 PVP and posterior 1/3 PVP models; (D) Maximum stress map of L3 vertebral body fracture, anterior 1/3 PVP, middle 1/3 PVP and posterior 1/3 PVP models; (E) Maximum response diagram of L2-3 intervertebral disc under 6 working conditions: L2 vertebral fracture, anterior 1/3 PVP, middle 1/3 PVP, and posterior 1/3 PVP models.

In the cement perfusion model, the distribution of cement exhibited variations in relation to the stress distribution within the vertebral body across 6 distinct loading conditions, as illustrated in Figure 5. Specifically, during forward flexion, extension, and left and right lateral bending, the maximum stress values observed in the anterior 1/3 of the vertebral body were found to be lower than those recorded in the middle and posterior 1/3 regions of the vertebral body. The maximum stress values for the anterior 1/3 were measured at 70.0, 49.0, 73.2, and 52.1 MPa; for the middle 1/3 at 87.6, 49.2, 77.4, and 60.1 MPa; and for the posterior 1/3 at 99.1, 49.2, 78.9, and 86.8 MPa, respectively. Under left and right rotation conditions, the maximum stress values of the middle 1/3 of the vertebral cement model were the smallest, 64.5 and 66.0 MPa, respectively (Fig. 6B)

### 3.3. Stress distribution and magnitude on adjacent vertebral bodies L1 and L3

**Figure 7.**
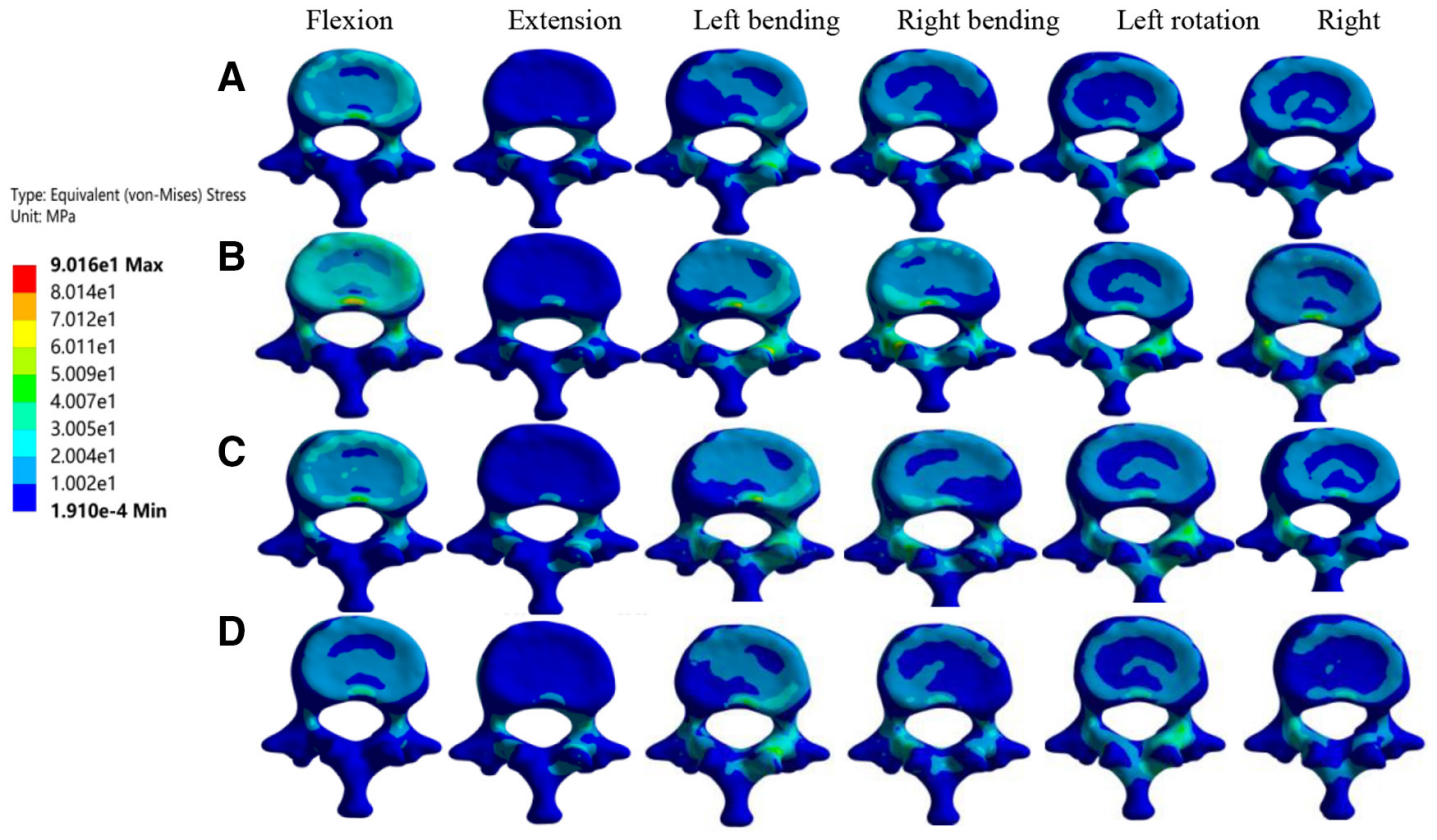
Stress distribution on adjacent L1 and L3 vertebral bodies in L2 vertebral fracture model and anterior 1/3 PVP, middle 1/3 PVP and posterior 1/3 PVP models. Stress distribution of L1 vertebral body after L2 vertebral fracture under 6 working conditions. (A) stress distribution of L1 vertebral body when no bone cement is perfused in L2 vertebral fracture; (B) stress distribution of L1 vertebral body when bone cement is perfused in the anterior 1/3 of L2 vertebral fracture; (C) stress distribution of L1 vertebral body when bone cement is perfused in the middle 1/3 of L2 vertebral fracture; (D) stress distribution of L1 vertebral body when bone cement is perfused in the posterior 1/3 of L2 vertebral fracture.

**Figure 8.**
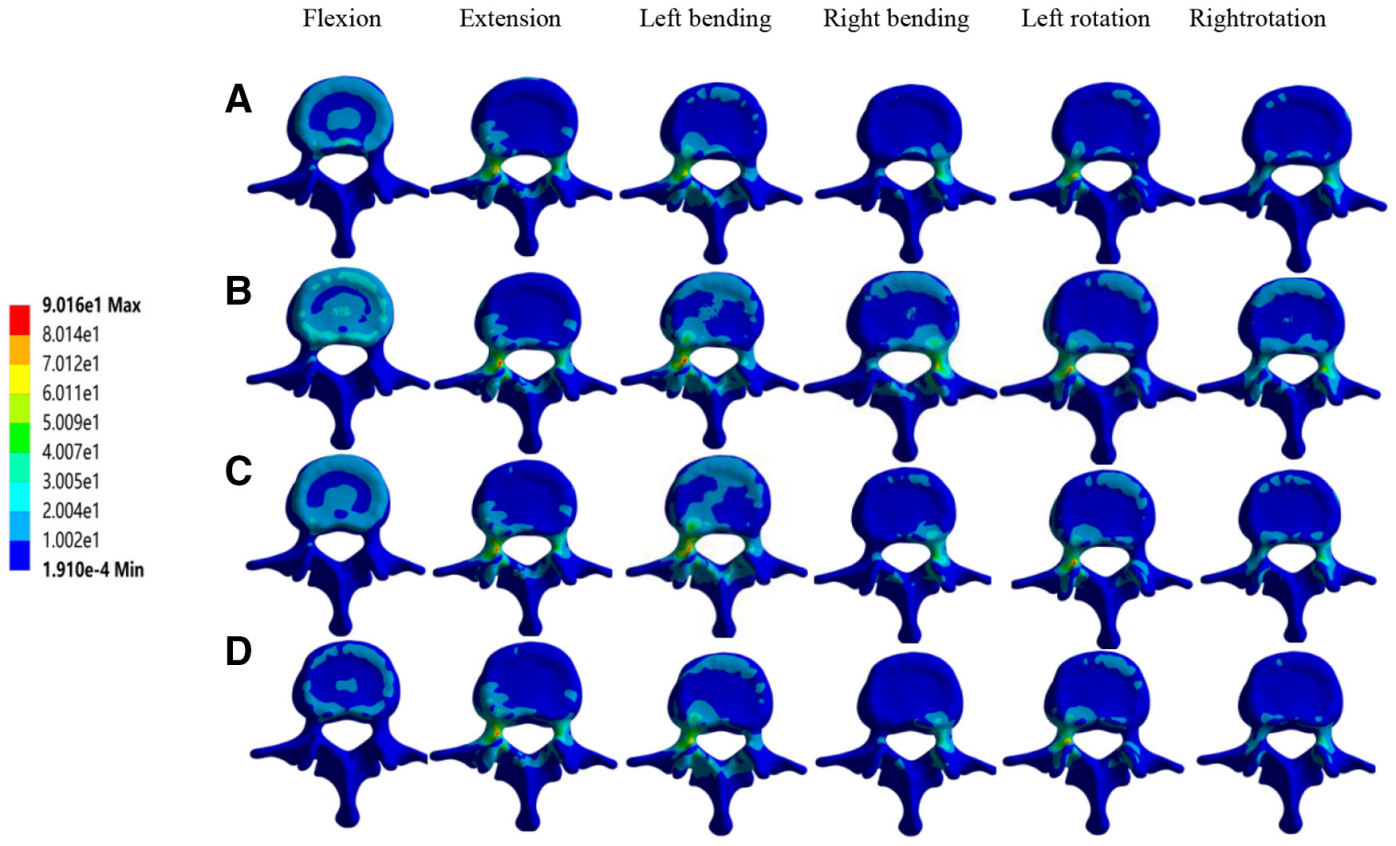
Stress distribution of L3 vertebral body after L2 vertebral fracture under 6 working conditions. (A) stress distribution of L3 vertebral body when no bone cement was perfused in L2 vertebral fracture; (B) stress distribution of L3 vertebral body when bone cement was perfused in the anterior 1/3 of L2 vertebral body; (C) stress distribution of L3 vertebral body when bone cement was perfused in the middle 1/3 of L2 vertebral body; (D) stress distribution of L3 vertebral body when bone cement was perfused in the posterior 1/3 of L2 vertebral body; (D) stress distribution of L3 vertebral body when bone cement was perfused in the posterior 1/3 of L2 vertebral body.

In the comparison of fracture models, the stress distribution of L1 vertebral body was more uniform under 6 loading conditions in the 3 models perfused with bone cement. In the 3 models perfused with bone cement, the maximum stress value of bone cement perfused into the posterior 1/3 of the L2 vertebral body on the L1 vertebral body increased significantly and approached the fracture model when the left side of the vertebral body was bent, and the maximum stress value of the 3 perfused bone cement models on the L1 vertebral body was close under the other 5 working conditions (Fig. 6A). Compared with the fracture model, the stress distribution of L3 vertebral body was more uniform in the 3 models perfused with bone cement under 6 working conditions (Figs. 7 and 8). There was no significant difference in the maximum stress value of L3 vertebral body between the fracture model and the perfused cement model when the spine was extended, bent on the right side and rotated on the right side (Fig. 6C). However, when the left side of the spine was bent and rotated, the maximum stress values of the fracture model and the posterior 1/3 of the L2 vertebral body model on the L3 vertebral body were close to and higher than those of the anterior and middle 1/3 of the L2 vertebral body cement models on the L3 vertebral body, and their values were 86.9 MPa, 96.3 MPa, 86.1 MPa, 91.0 MPa, 67.8 MPa, 73.5 MPa, 79.2 MPa, and 74.6 MPa, respectively.

### 3.4. Maximum von-Mises stress distribution and size on L1-2 disc and L2-3 disc

**Figure 9.**
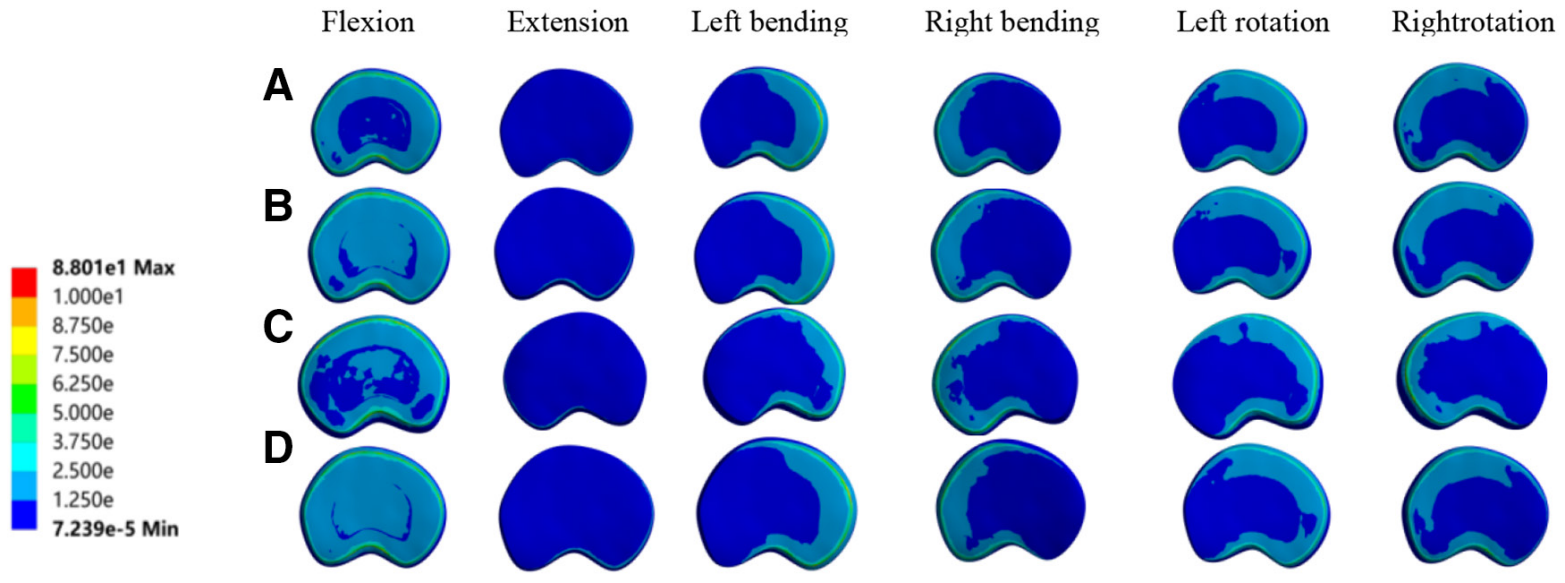
Von- Mises stress cloud diagram of L1-2 intervertebral disc under 6 working conditions. (A) stress distribution of L1-2 intervertebral disc when bone cement is not perfused in L2 vertebral fracture; (B) stress distribution of L1-2 intervertebral disc when bone cement is perfused in anterior 1/3 of L2 vertebral fracture; (C) stress distribution of L1-2 intervertebral disc when bone cement is perfused in middle 1/3 of L2 vertebral fracture; (D) stress distribution of L1-2 intervertebral disc when bone cement is perfused in posterior 1/3 of L2 vertebral fracture.

**Figure 10.**
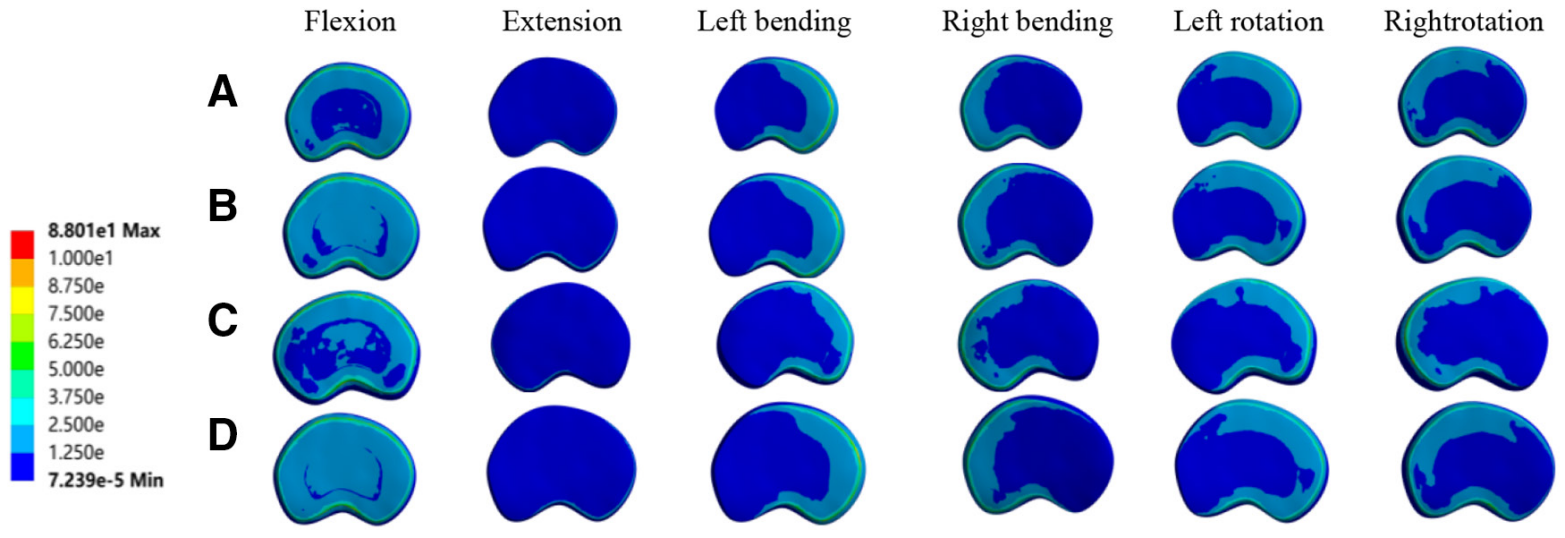
Von-Mises stress cloud diagram of L1-2 intervertebral disc under 6 working conditions. (A) stress distribution of L1-2 intervertebral disc when bone cement is not perfused in L2 vertebral fracture; (B) stress distribution of L1-2 intervertebral disc when bone cement is perfused in anterior 1/3 of L2 vertebral fracture; (C) stress distribution of L1-2 intervertebral disc when bone cement is perfused in middle 1/3 of L2 vertebral fracture; (D) stress distribution of L1-2 intervertebral disc when bone cement is perfused in posterior 1/3 of L2 vertebral fracture.

The stress distribution on the L1–2 disc and L2–3 disc did not change significantly in the L2 vertebral fracture model, the anterior, middle, and posterior 1/3 PVP models of the L2 vertebral fracture (Figs. 9 and 10).10). However, in the case of extension loading, the L2 vertebral fracture model produced lower maximum stress values to the L1–2 disc than the instilled cement model. In the other 5 working conditions, there was no significant difference in the maximum stress values generated for the L1–2 disc (Fig. 6D). For the L2–3 disc, the vertebral body showed a difference in the maximum stress value produced by the fracture model compared with the perfused cement model group only in the right lateral bending; there was no significant difference in the maximum stress value produced by the L2–3 disc under the other 5 working conditions (Fig. 6E).

### 3.5. Maximum equivalent elastic strain of L2 vertebral body

**Figure 11.**
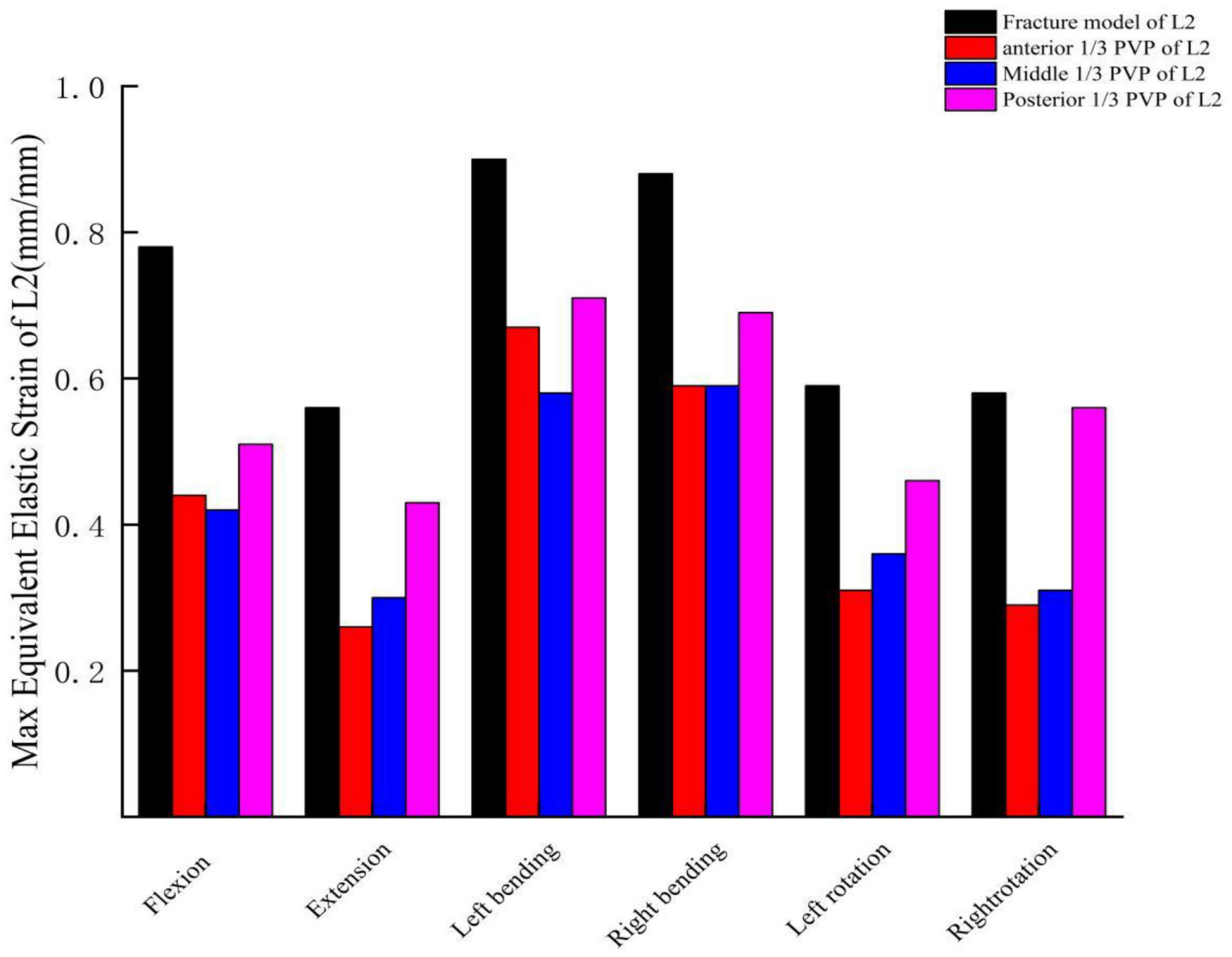
Maximum equivalent elastic strain of L2 vertebral body under 6 working conditions. Strain is a measure of deformation and describes the degree of deformation of an object. Strain represents the relative change in length, is the ratio of the shape variable to the original length dimension, and is represented by the mathematical symbol ε. ε = ΔL/L. where ΔL refers to the length change caused by the deformation (stretching or compression) when the linear object is subjected to external force, and L refers to the initial length of the linear object. Therefore, we can see that when bone cement is perfused into the anterior middle of the vertebral body, the stress variables produced by the vertebral body are the smallest, the morphological changes of the trabecular microarchitecture of the vertebral body are the smallest, and then the stress variables produced by bone cement perfused into the posterior 1/3 of the vertebral body are relatively large, resulting in greater compression or tensile changes produced by the microarchitecture of the vertebral body and easily leading to increased internal structure destruction.

The maximum strain of L2 vertebral fracture model was higher than that of perfusion cement group under 6 working conditions, and the maximum strain variables of forward flexion, extension, left and right lateral bending and left and right rotation were 0.78, 0.56, 0.9, 0.88, 0.59, and 0.58 mm/mm, respectively. However, in the 3 cemented model groups, the maximum dependent variables of L2 vertebral body under the 6 conditions of anterior, middle, and posterior 1/3 PVP models of L2 vertebral body were 0.44, 0.26, 0.67, 0.59, 0.31, 0.29 mm/mm, 0.42, 0.3, 0.58, 0.59, 0.36, 0.31 mm/mm, 0.51, 0.43, 0.71, 0.69, 0.46, and 0.56 mm/mm/mm, respectively; the maximum dependent variables of anterior and middle 1/3 cemented models of L2 vertebral body were similar and smaller than those of posterior 1/3 cemented models of L2 vertebral body (Fig. 11).

### 3.6. Maximum deformation displacement of L2 vertebral body

**Figure 12.**
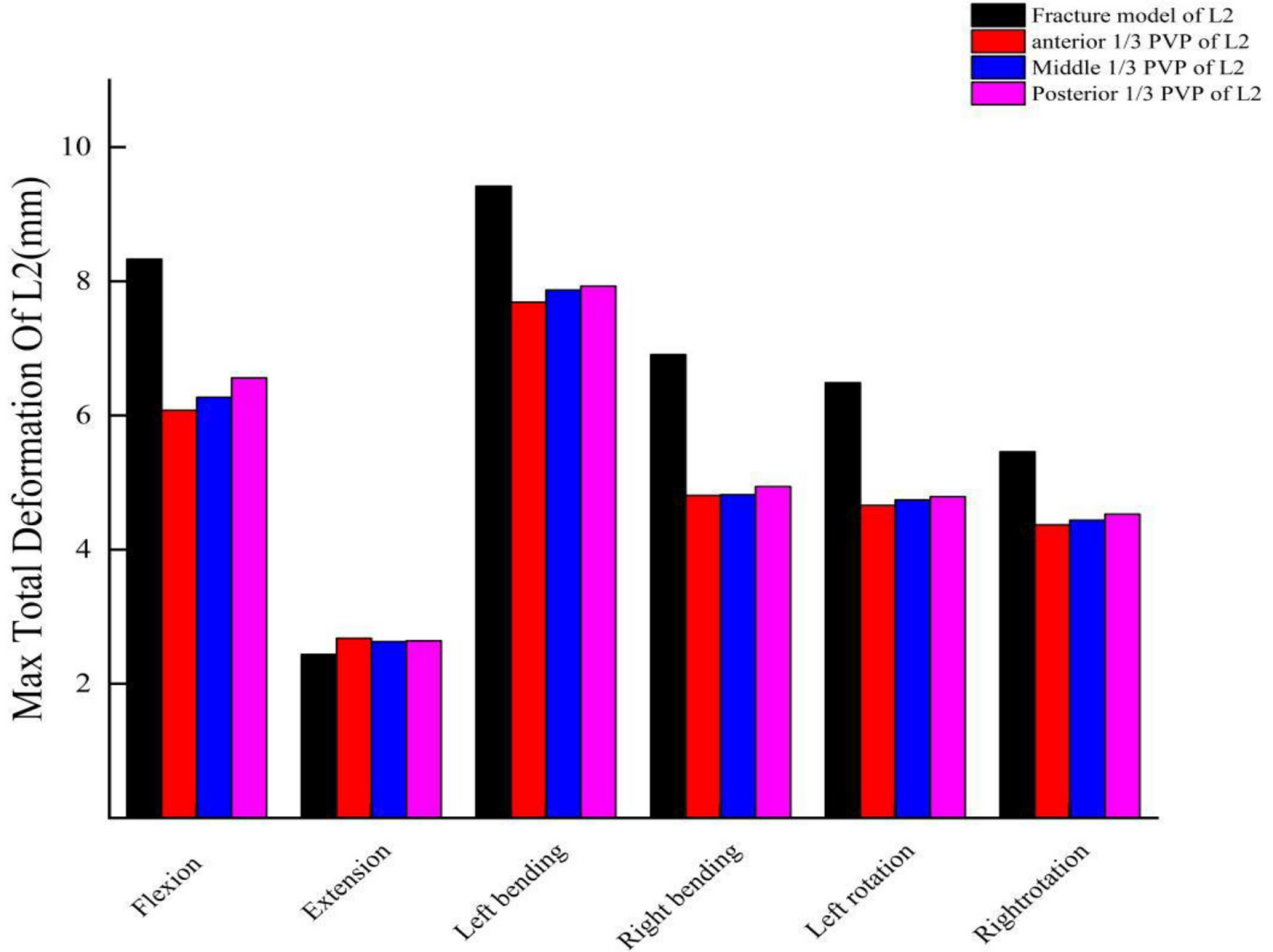
Maximum deformation displacement of L2 vertebral body under 6 working conditions

Under forward flexion, left and right lateral bending and left and right rotation conditions, the deformation displacement of L2 vertebral fracture unperfused cement model was significantly greater than that of perfused cement model group, and the maximum dependent variables were 8.33, 9.42, 6.91, 6.49, and 5.46 mm, respectively. In the 3 models perfused with bone cement groups, the maximum deformation displacements of the L2 vertebral body were 6.08, 7.69, 4.81, 4.66, 4.37 mm, 6.27, 7.87, 4.82, 4.74, 4.44 mm, 6.56, 7.93, 4.94, 4.79, and 4.53 mm in the anterior, middle, and posterior 1/3 PVP models of the L2 vertebral body under 5 working conditions: forward flexion, left and right lateral bending, and left and right rotation, respectively; the maximum deformation displacements of the anterior and middle 1/3 cement models of the L2 vertebral body were similar and smaller than those of the posterior 1/3 perfusion models of the L2 vertebral body, but the advantages were not significant. (Fig. 12).

## 4. Discussion

The emphasis in the management of osteoporotic vertebral compression fractures has shifted from the mere completion of surgery to the execution of an efficacious surgical procedure. An effective surgical approach necessitates considerations of adequate perfusion and reasonable modulus of elasticity.^[12,13]^ Nevertheless, there remains a paucity of compelling research findings regarding the impact of bone cement distribution on the affected and neighboring vertebrae. Furthermore, given the non-degradability and high biomechanical stress of polymethylmethacrylate bone cement, a more extensively filled and widely distributed application may not necessarily represent the optimal choice for this procedure.^[7–9]^ How to reduce the risk of adjacent vertebral fractures and enhance vertebral re-collapse after PVP is very worth studying.

Retrospective studies have pointed out that the distribution, diffusion, and leakage of bone cement may be the cause of refracture, and the text of these studies shows the distribution of bone cement in the central mass, poor diffusion, thoracolumbar stress concentration areas, the distribution of bone cement in the posterior column and nonfinal plate-like diffusion, and intradiscal leakage of bone cement in some cases.^[28–30]^ In this study, we confirmed that inadequate cement diffusion through the mechanics of the L2 vertebral body will lead to uneven stress on the adjacent vertebral body and the injured vertebra. When bone cement is distributed in the anterior middle of vertebral body, vertebral stress and stress variables are more evenly distributed than bone cement in the posterior column. These mechanical inconsistencies cause fractures of the vertebral body, which are strongly mechanically proven. Each kind of tissue has its elastic modulus, and the higher the value of elastic modulus, the greater the stress that causes certain elastic deformation of the material. The elastic modulus of bone cement is 3000 MPa and 34 MPa for cancellous bone of osteoporotic vertebral bodies,^[18,19]^ and the elastic modulus of bone cement is much higher than that of cancellous bone of osteoporotic vertebral bodies. In this study, after simulated injection of bone cement into the L2 vertebral body, the stiffness of the cement distribution area increased significantly compared with the surrounding, and the pressure on the cement less area decreased.^[31]^ By simulating the different distribution positions of bone cement, we found that when the bone cement was distributed in the anteromedial part of the vertebral body, the stress, strain value and deformation displacement produced by the fractured vertebral body and adjacent vertebral body were relatively small, so it was speculated that the elastic modulus of the vertebral body increased when the bone cement was distributed in the anteromedial part of the vertebral body, shared the conduction of stress concentration, and restored the partial height of the vertebral body, which may allow the pressure borne by the vertebral body to be transmitted to the upper and lower lumbar spine through the bone cement, thereby reducing the pressure of the cortical bone and cancellous bone in the central area and protecting the weak area of the vertebral center, thus possibly reducing the risk of refracture of the injured vertebra and adjacent vertebral fractures.

The spine comprises the physiological activities of the spine within a certain range of forward flexion, extension, left and right flexion, and left and right rotation.^[32]^ The etiology of osteoporotic vertebral compression fractures is generally nonviolent and may be caused by any activity of the spine, and more patients in clinical practice present with increased lumbar or back pain during postural changes, and the external forces causing fractures are not described.^[33]^ In this study, the L2 vertebral body is located in the turning zone of the thoracolumbar and lower lumbar segments, and when the external force bursts, this is the mechanical intersection area, which is more reflective of the real situation of the force, and the L2 vertebral body is well represented as the fracture object.^[34,35]^ According to the maximum stress of the vertebral body, the maximum stress value of the cement model in the anterior 1/3 of the L2 vertebral body was reduced by 19% and 29% compared with the cement model in the middle and posterior 1/3 of the vertebral body during forward flexion, by 5% and 6% during left bending, and by 13% and 39% during right bending. When simulating left and right rotation, the maximum stress value of the cement model in the middle 1/3 of the vertebral body was the smallest, so the perfusion in the first 1/3 was more effective. In the left lateral curvature of the spine, the maximum stress value of the L1 vertebral body produced by the perfusion of bone cement in the posterior 1/3 of the L2 vertebral body was significantly increased and close to that of the fracture model, and the maximum stress value of the L1 vertebral body was similar in the 3 cement models at the other 5 working conditions (Fig. 6A), and the fracture was more likely to occur when the bone cement was perfused in the posterior 1/3.In the left spinal curvature and left rotation, the maximum stress value produced by the fracture model and the posterior 1/3 of the vertebral body cement model on the L3 vertebral body was close to and significantly higher than that produced by the anterior and middle 1/3 of the vertebral body perfusion cement model on the L3 vertebral body (Fig. 6C), so it is not recommended to inject the bone cement into the posterior 1/3 of the vertebral body in clinical practice, and simple posterior 1/3 perfusion can be considered as ineffective perfusion.

In this study, when the maximum deformation displacement of L2 vertebral body occurred, the maximum displacement of unperfused cement model was significantly higher than that of fracture-perfused cement model (Fig. 12), further confirming the reliability of PVP in the treatment of osteoporotic vertebral compression fractures, and vertebroplasty may be a better choice in the thoracolumbar junction area at an early stage. At the maximum elastic strain of L2 vertebral body, the maximum strain of anterior and middle 1/3 bone cement models of vertebral body was similar, which was smaller than that of posterior 1/3 bone cement model of L2 vertebral body, while the maximum deformation displacement of bone cement perfused into anterior and middle vertebral body was slightly superior to that of posterior bone cement perfused under 6 working conditions (6.08, 6.27 vs 6.56 mm; 7.69, 7.87 vs 7.93 mm; 4.81, 4.82 vs 4.94 mm; 4.66, 4.74 vs 4.79 mm; 4.37, 4.44 vs 4.53 mm), and this result indicated that good maximum elastic strain could be obtained for anterior and middle 1/3 bone cement perfused, and the anterior and middle 1/3 bone cement perfused may be the best choice in clinical work (Fig. 11). For the center of the vertebral body that is most likely to collapse.^[36]^ We concluded that when the bone cement is distributed in the anteromedial part of the vertebral body, the stress, strain value and deformation displacement produced by the fractured vertebral body and adjacent vertebral body are small, so the concentrated conduction of the stress is shared, which may restore the height of the part of the vertebral body, so that the pressure borne by the vertebral body is transmitted to the upper and lower lumbar vertebrae through the bone cement, reduce the pressure of the cortical bone and cancellous bone in the central area, protect the weak area of the vertebral body center, and reduce the risk of the injured vertebra and adjacent vertebral fractures.

In this study, because the shape of the postoperative vertebral bone cement was not exactly the same and the real events could not be simulated, we tried to perform finite element analysis using real bone cement. However, the influence of cement shape on the experimental results cannot be controlled. We therefore used a cylindrical cement model, which is easy to calculate by computer and ensures the reproducibility of the study. Second, the FEM is oversimplified and does not simulate muscle, fascia, blood vessels, skin, multiple vertebral fractures, spinal deformities, etc, while all these factors may affect our results. Therefore, our study only serves as a reminder and cannot completely reflect the clinical efficacy and biomechanical changes.

## Author contributions

**Software:** Changbing Wu.

**Validation:** Changbing Wu.

**Investigation:** Jie Liu.

**Conceptualization:** Hao Long, ZhuBo He, Fu Yong, GuoXian Wang, Ge Bing.

**Formal analysis:** Hai Tao Gao.

**Visualization:** Hai Tao Gao.

**Writing – original draft:** Changbing Wu.

**Writing – review & editing:** Changbing Wu, Jie Liu.
